# The Ross Procedure: Imaging, Outcomes and Future Directions in Aortic Valve Replacement

**DOI:** 10.3390/jcm13020630

**Published:** 2024-01-22

**Authors:** Domenico Galzerano, Naji Kholaif, Bandar Al Amro, Mohammed Al Admawi, Abdalla Eltayeb, Amal Alshammari, Giovanni Di Salvo, Zohair Y. Al-Halees

**Affiliations:** 1 Heart Center Department, King Faisal Specialist Hospital and Research Center, Riyadh 11564, Saudi Arabia; domenicogalzerano@libero.it (D.G.); b.alamro@hotmail.com (B.A.A.); al-admawi@hotmail.com (M.A.A.); abdullaheltayeb2002@gmail.com (A.E.); amalmubarak888@gmail.com (A.A.); alhalees@kfshrc.edu.sa (Z.Y.A.-H.); 2 College of Medicine, Alfaisal University, Riyadh 11533, Saudi Arabia; 3 Department of Cardiac, Thoracic, Vascular Sciences and Public Health, Medical School, University of Padua, 35122 Padua, Italy; giodisal@yahoo.it

**Keywords:** Ross procedure, prosthetic aortic valve, aortic valve replacement, valvular heart disease, adverse valve-related events, surgical techniques, valve imaging, valve hemodynamics, contemporary surgical techniques and innovations, tissue engineering

## Abstract

The Ross procedure is gaining recognition as a significant option for aortic valve replacement (AVR), and is particularly beneficial in specific patient groups. Although categorized as a class IIb recommendation in the 2020 American College of Cardiology (ACC)/American Heart Association (AHA), and the European Society of Cardiology (ESC) management guidelines on valvular heart disease, recent studies bolster its credibility. Research, including a propensity-matched study, underlines the Ross procedure’s association with enhanced long-term survival and reduced adverse valve-related events compared to other AVR types. This positions the Ross procedure as a primary option for AVR in young and middle-aged adults within specialized centers, and potentially the only choice for children and infants requiring AVR. This review meticulously examines the Ross procedure, covering historical perspectives, surgical techniques, imaging, and outcomes, including hemodynamic performance and quality of life, especially focusing on pediatric and young adult patients. It explores contemporary techniques and innovations like minimally invasive approaches and tissue engineering, underscoring ongoing research and future directions. A summarization of comparative studies and meta-analyses reiterates the Ross procedure’s superior long-term outcomes, valve durability, and preservation of the left ventricular function, accentuating the crucial role of patient selection and risk stratification, and pinpointing areas for future research.

## 1. Introduction

The Ross procedure, also known as pulmonary autograft replacement of the aortic valve, is a surgical technique by which the patient’s diseased valve is replaced by their own pulmonary valve (autograft). The used pulmonary valve is replaced by a pulmonary allograft or other suitable valve prosthesis. The rationale behind this procedure stems from the many advantages it offers. These include superior durability, better hemodynamics than any other aortic valve substitute, the potential for growth in younger patients and not requiring anticoagalation therapy with its well-known limitations. This translates into improved long-term outcomes and better quality of life [1,2].

The Ross procedure has gained significant attention as an alternative to other aortic value substitutes, particularly in young and active patients. The growth potential of the autograft valve makes it an attractive option for pediatric patents including infants and neonates requiring aortic valve replacement, potentially avoiding the need for repeat surgeries [3,4]. However, the Ross procedure is not without limitations. It is technically a demanding surgery that requires a skilled surgical team. It is associated with longer aortic cross-clamp and operative times compared to other aortic valve replacement procedures. Additionally, there is the risk of a replaced pulmonary valve dysfunction long-term, potentially necessitating a reoperation or percutaneous reintervention in some patients [5]. As a result of these concerns and despite the growing body of evidence showing the excellent long-term outcomes, the Ross procedure remains a class IIb recommendation in the most recent 2020 ACC/AHA and 2021 ESC guidelines on valvular heart disease [6,7].

The Ross procedure’s usefulness has gained renewed interest after the publication of many studies including a propensity-matched study showing that it is associated with better long-term survival and freedom from adverse valve-related events compated to bioprosthetic or mechanical AVR. Therefore, in specialized cardiac centers with expertise, the Ross procedure can be considered the optimal option for adult and middle-aged adults undergoing AVR [8,9]. Needless to say, it may be the only option for infants and neonates requiring AVR for non-repairable aortic valve disease [4].

By examining the existing literature and synthesizing the available evidence, this review aims to shed light on the benefits, limitations, and appropriate patient selection for the Ross procedure. We hope that this will ultimately assist clinicians in making informed decisions regarding the utilization of the procedure in their clinical practice.

## 2. Historical Perspective

### 2.1. Overview of the Development and Evolution of the Ross Procedure

The Ross procedure, named after its pioneering surgeon Donald Ross, has undergone significant development and evolution since its inception. It was first introduced in the 1960s as a novel technique for aortic valve replacement using the patient’s own pulmonary valve. In his original paper describing the technique, Ross also suggested that the pulmonary autograft can be used to replace the mitral valve as well (later referred to as the Ross II procedure) [10,11]. Over the years, advancements in surgical techniques, perioperative management, and excellent long-term outcomes have led to considerable interest in the procedure. Initially, it was primarily offered to adults and older patients with various aortic valve pathologies, including patients with rheumatic aortic valve disease manifesting as aortic valve regurgitation/stenosis or both [12]. Its application was expanded to include younger patients with congenital aortic valve disease and metabolic diseases such as familial hypercholesterolemia [13].

### 2.2. Milestones and Key Contributors in Advancing the Technique

Several milestones and key contributors have played a significant role in advancing the Ross procedure. Donald Ross first described the technique and performed the initial series of surgeries [10]. His pioneering work laid the foundation for the procedure and demonstrated the potential benefits of using the patient’s own pulmonary valve to replace the aortic or mitral valve. Subsequently, other cardiac surgeons worldwide further refined the surgical technique, expanding its indications and improving outcomes. The emphasis was on AVR. Mitral valve replacement using the pulmonary valve (Ross II) did not gain as much attention [14].

Notable contributors include Dr. Magdi Yacoub, who contributed to the advancements in the Ross procedure and its widespread adoption. His work included the only randomized controlled trial comparing Ross with a homograft aortic root replacement in adults with aortic valve disease, demonstrating the superiority of the Ross procedure [15]. It took about 20 years for the Ross procedure to catch on in the United States and Canada. But then, North American surgeons including Ronald Elkins, Paul Stelzer, Tirone David, John Oswalt, and many others made significant contributions to the understanding and refinement of the Ross procedure [16,17]. Zohair Al Halees, who performed one of the largest series worldwide, highlighted the importance of candidate selection in the long-term results of the Ross procedure, emphasizing the need for careful patient selection to achieve optimal outcomes [18].

## 3. Surgical Technique

The Ross procedure involves several key steps. First, the patient is placed on the cardiopulmonary bypass and the aorta is cross-clamped and cardioplegia is given. The aortic valve is then carefully examined and if not repairable is excised and the aortic annulus is sized. There should not be much discrepancy in size between the aortic and the pulmonary annuli. Not more than a 2–3 mm discrepancy should be accepted. Very little RV muscle is kept below the pulmonary valve and only a few mms of the pulmonary artery are kept above pulmonary valve commissures. The pulmonary valve is then sutured to the aortic annulus, creating a new normally functioning neoaortic valve. There are several techniques to implant the autograft However, the technique of a freestanding aortic root with a coronary transfer is the most commonly used today. Subsequently, a pulmonary allograft or a prosthetic valve is implanted in the pulmonary position. Finally, the aorta is unclamped, and the patient is weaned off a cardiopulmonary bypass [19].

### Surgeon’s Expertise

The Ross procedure necessitates a high level of surgical expertise due to its complexity and technical demands. Surgeons must possess specialized experience in aortic root surgery, as the techniques required for the Ross procedure are similar to those used in complex root surgeries, such as familiarity with root anatomy and replacement as well as homograft root replacement. In centers with experience of and a high volume in performing the Ross procedure, long-term survival and freedom from valve-related complications are better than alternative procedures in young and middle-aged patients [20].

## 4. Patient Selection Criteria

Patient selection for the Ross procedure requires careful consideration. The procedure is most suitable for younger patients with aortic valve disease, in whom stenosis is the predominant hemodynamic manifestation, particularly those who desire freedom from lifelong anticoagulation therapy or who are engaged in high-impact physical activities [21]. Patients with a rheumatic aortic valve disease should be carefully assessed as it has been demonstrated that the pulmonary autograft becomes susceptible to rheumatic fever [22]. In general, patients with severe aortic valve regurgitation and with a dilated aortic root of more than 27–28 mm may not be as good candidates as those with aortic stenosis and normal aortic roots [23]. Additional measures to stabilize the aortic root and prevent progressive dilatation need to be undertaken. Patients with connective tissue disorders such as Marfan and Loeys–Dietz syndrome are not good candidates for the procedures [24].

Candidates should have a normally functioning pulmonary valve without a significant pathology. It is not advisable to use a bicuspid or a quadricuspid pulmonary valve or a pulmonary valve with too many large fenestrations [19].

The Ross procedure may not be suitable for older patients and those with significant comorbidities, as they may have a higher risk of complications. The procedure may still be an option in patients with combined mitral and aortic valve disease if a good mitral valve repair can be accomplished [25].

Therefore, careful patient selection and consideration of the procedure’s technical challenges and potential drawbacks are crucial factors in achieving optimal outcomes.

## 5. Outcomes and Complications

### 5.1. Analysis of Short-Term and Long-Term Survival Rates

The Ross procedure has demonstrated favorable short-term and long-term survival rates in various studies. Regarding short-term outcomes, several studies have reported low operative mortality rates ranging from 0% to 4% [25]. Long-term survival rates after the Ross procedure have also been encouraging. Long-term outcomes showed 10-year survival rates ranging from 81% to 94%, while 20-year survival rates ranged from 65% to 92% [26]. These results suggest that the Ross procedure can provide durable survival benefits for appropriately selected patients. In a propensity-matched study, the Ross procedure demonstrated conferring a survival advantage when compared with mechanical valve replacement [27]. In a more recent study, the procedure was associated with better long-term survival when compared to patients with mechanical or bioprosthetic AVR. Therefore, in cardiac centers with expertise, the Ross procedure is considered the primary option for young and middle-aged adults undergoing AVR [28]. Table 1 summarizes the short- and long-term outcomes of the Ross procedure.

### 5.2. Evaluation of Postoperative Complications and Their Management

**Figure 1:**
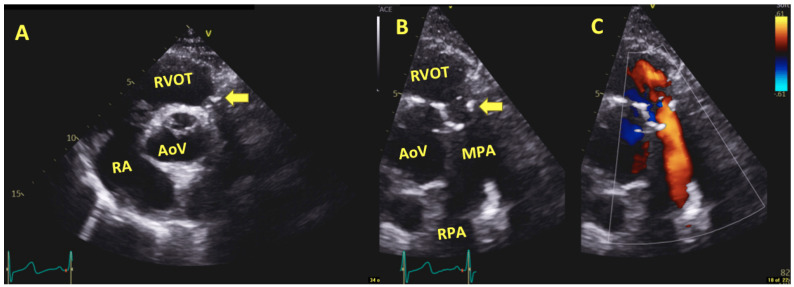
Transthoracic echocardiography Parasternal short axis view; Panel (**A**): Degenerative homograft cusps (yellow arrow, diastolic phase); Panel (**B**): Degenerative homograft cusps (yellow arrow, systolic phase); Panel (**C**): color Doppler showing the regurgitation originating from the right pulmonary artery suggestive of severe regurgitation; Abbreviations: RVOT: Right ventricular outflow track, RA: Right atrium, AoV: Aortic valve, MPA: Main trunk of pulmonary artery, RPA: Right main branch of pulmonary artery.

**Figure 2:**
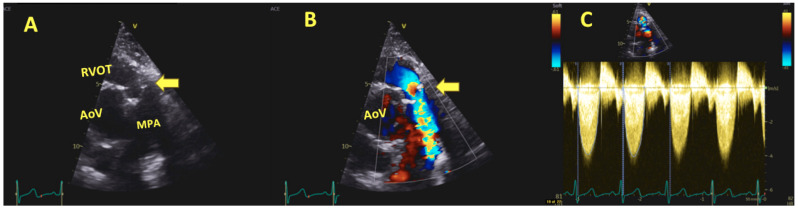
Panel (**A**) Parasternal short-axis (PSAX) view at aortic valve level short-axis (PSAX) view showing severe homograft stenosis with degenerative homograft cusps (yellow arrow) RVOT and MPA. Panel (**B**); color Doppler demonstrating systolic flow acceleration starting at the level of pulmonic homograft (yellow arrow) indicating pulmonic valve stenosis (PS). Panel (**C**); Continuous-flow (CW) Doppler across the pulmonic valve showing severe PS with peak and mean gradients of 69 and 42 mmHg, respectively; Abbreviations: AoV: aortic valve, RVOT: right ventricle out-flow tract, MPA: main pulmonary artery.

**Figure 3:**
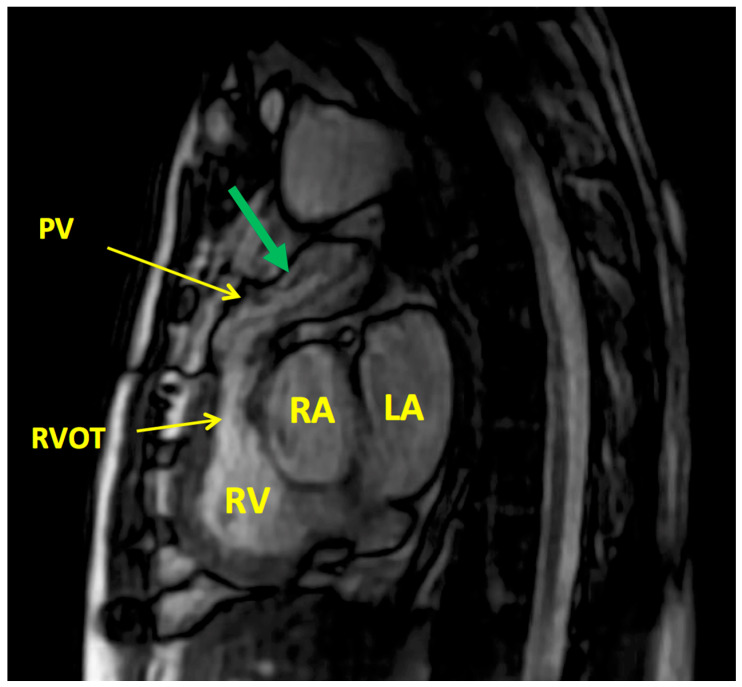
Cardiac magnetic resonance (CMR) with oblique sagittal stack cine cuts at different level showing pulmonic homograft stenosis. Green arrow showing defacing (showing significant stenosis); Abbreviations: PV: pulmonary valve, RVOT: right ventricular outflow track, RA: right atrium. RV: right ventricle, LA: left atrium.

Early complications include technical issues causing autograft malfunction, bleeding, infection, arrhythmias, and coronary artery problems leading to left ventricular dysfunction. Later complications include valve-related issues, such as autograft dysfunction and/or pulmonary valve substitute dysfunction. Autograft dysfunction can occur due to valve regurgitation, stenosis or both or due to annular or aortic root dilatation. Progressive aortic root dilatation was observed frequently in the early series, particularly in young adults (Figure 1 and Figure 2). This actually at one stage dampened the enthusiasm for the procedure [29]. Strict blood pressure control to avoid hypertension particularly early is very important in avoiding this complication. Techniques to prevent this were introduced, including aortic root reinforcement ± sino-tubular junction reinforcement or placing the autograft inside a cylinder Dacron vascular graft with or without sinuses of Valsalva (Figure 3) [30,31].

**Figure 4:**
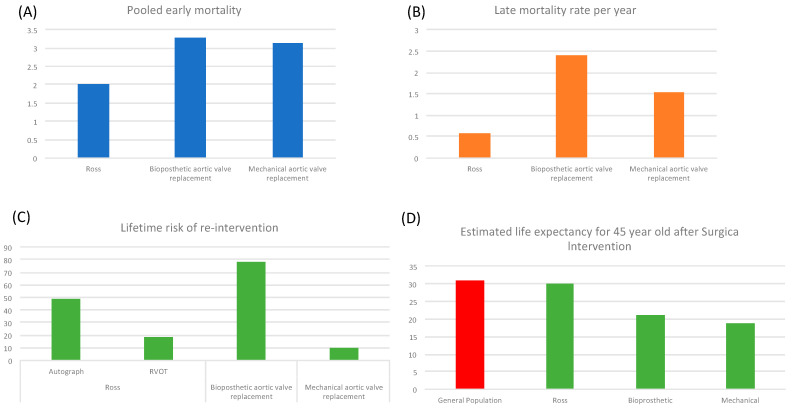
This figure presents a systematic review encompassing three distinct patient populations from studies reporting outcomes in adult patients aged between 18 and 55 years who have undergone the Ross procedure [34], bioprosthetic aortic valve replacement [35], and mechanical aortic valve replacement [36]. Panel (**A**) illustrates the aggregated early mortality rates, represented on the Y-axis, across different cohorts subjected to the three aforementioned surgical procedures. Panel (**B**) displays the aggregated annual late mortality rates as percentages. Panel (**C**) delineates the lifetime risk percentages for re-intervention in the Ross procedure, with separate data for the autograft in the aortic position and right ventricular outflow tract (RVOT) surgical interventions, as well as for bioprosthetic and mechanical aortic valve replacements. Panel (**D**) involves pooled data input into a microsimulation model, estimating the life expectancy, in years, of a 45-year-old patient following different aortic valve replacement surgeries. These estimates are compared (highlighted in red) with the projected life expectancy, post-45 years, of the general population that has not undergone any surgical interventions.

Recently, a personalized external aortic root support (PEARS), a custom-made macroporous mesh, was used to stabilize a dilated aortic root (Exstent limited^®^). This approach was adapted to the Ross procedure (ROSS-PEARS) (Figure 4) [32]. However, the long-term outcome of such modifications is yet to be validated. [33]. Pulmonary valve substitute dysfunction can be stenosis, regurgitation, or both. This remains the weak link in the procedure to the extent that some call it “turning a single-valve disease into a double-valve disease”. The management of this complication may require surgical or percutaneous intervention. The increased interventional cardiologists’ experience and the availability of many percutaneous pulmonary valve substitutes reduced apprehension about this potential problem.

The autograft must function perfectly or “unfortunately the patient will end up with a different valve in the aortic position and lose the original pulmonary valve to a substitute requiring life-long surveillance” [37]. Valve sparing reoperations are possible for failed pulmonary autografts. Although this depends on the mechanism of autograft failure, still up to 50% salvage rates can be achieved in experienced hands [38].

### 5.3. Comparison of Outcomes with Alternative Procedures

When comparing the outcomes of the Ross procedure with alternative procedures, such as a mechanical valve, bioprosthetic or homograft aortic root replacement, several factors need to be considered. A mechanical valve replacement provides excellent durability but requires lifelong anticoagulation therapy, which may increase the risk of bleeding complications. A bioprosthetic AVR or homograft aortic root replacement avoids the need for anticoagulation therapy but has limited durability due to degeneration. The younger the patient is, the faster will be the degeneration and need for re-intervention.

Comparative studies have shown similar or improved survival rates and lower rates of valve-related complications with the Ross procedure compared to other aortic valve replacement options. One study comparing the Ross procedure to a mechanical valve replacement in young patients demonstrated similar long-term survival rates but significantly lower rates of reoperation in the Ross group [39]. Another study comparing the Ross procedure to a homograft aortic root replacement found comparable long-term survival rates but a higher incidence of valve-related complications in the homograft group [15].

In propensity-matched studies, the Ross procedure was associated with better long-term survival and freedom from adverse valve-related events compared with mechanical or bioprosthesis AVR [9].

Today, dealing with the durability, when comparing the Ross procedure with the bioprosthetic valve replacement, we have to take in consideration the possibility of treating the bioprosthetic valve dysfunction by a transcatheter valve in the procedure. It is true that this is not a surgical reintervention but nevertheless another procedure with an incurred cost. At present, a valve in valve therapy is not an option for a failed autograft.

In terms of quality of life, the Ross procedure has been shown to provide better functional outcomes and higher patient satisfaction compared to a mechanical valve replacement, likely due to the avoidance of anticoagulation therapy and an optimal autograft function. Additionally, the Ross procedure has been associated with lower rates of thromboembolic events compared to a mechanical valve replacement. This translates to a better survival [40].

It is important to note that the choice of procedure should be individualized based on patient characteristics, including age, comorbidities, lifestyle, and surgeon expertise. Long-term follow-up and further research are necessary to continue evaluating and comparing the outcomes of the Ross procedure with alternative procedures in different patient populations. It is therefore imperative to consider the creation of Ross centers of excellence for achieving the best results.

### 5.4. Comparison of Outcomes with Alternative Procedures Entered in Microsimulation

Etnel and colleagues performed an extensive meta-analysis and systematic review focusing on patients younger than 55 years who underwent the Ross procedure (Figure 4) [34], bioprosthetic aortic valve replacement [35], and mechanical aortic valve replacement [36]. The rates of various events were compiled and fed into a microsimulation model, which was utilized to calculate both the life expectancy and the aggregate rate of events over a lifetime. Observations indicated that patients undergoing the Ross procedure exhibited a reduced incidence of perioperative mortality. Comparative analysis suggested more favorable long-term outcomes for the Ross procedure compared to a bioprosthetic or mechanical aortic valve replacement. When incorporated into the microsimulation, the probability of requiring re-operation over a lifetime was found to be low, and the projected life expectancy aligned closely with that of the general population.

## 6. Imaging in Ross Procedure

The Ross procedure imaging scenario necessitates a dedicated professional figure with specific expertise in this procedure. Multimodality imaging is paramount in the Ross procedure. As its clinical application grows, knowledge of the various imaging modalities used is required for the imager and beneficial for the interventional and surgical teams. The purpose of this review is to describe the key steps of the procedural imaging pathway.

### 6.1. Pre-Procedural Imaging

Pre-procedure cardiac multimodality imaging in the candidate selection, screening and planning for the Ross procedure is the guidelines recommended pathway in valvular and congenital heart disease. It includes a multimodality comprehensive assessment of cardiac pathologies and functions and associated great vessels anomalies like patent ductus arteriosus and coarctation of the aorta, which are sometimes challenging in the clinical arena [18,41,42]. It can determine patient eligibility based on anatomic features and measurements, provide measurements for appropriate homograft sizing, predict the risks of potential procedural complications and their likelihood of success. In this phase, the whole imaging armamentarium plays a role. In sizing, the AV and PV annulus, echocardiography represents the technique of choice even though cardiac CT allows a more precise sizing.

### 6.2. Intraoperative Imaging

Intraoperative imaging includes the use of transesophageal echocardiography able to confirm the preprocedural features and postoperatively detect the normal functioning of both the autograft and homograft as well as the ventricular function.

Multimodality imaging follow-up evaluations, including echocardiography and magnetic resonance imaging (MRI), allow early detection of any potential issues, optimizing reintervention decision-making and improving long-term outcomes.

### 6.3. Post-Operative and Long-Term Follow-Up Imaging

In the follow-up imaging pathway, echocardiography represents the first step. It is able to diagnose and follow over time the autograph and homograft behavior as well as the cardiac function. However, MRI and cardiac computed tomography are paramount tools in the imaging pathway in order to better assess the right ventricular function, the degree of the regurgitation as well as assessing both the coronary arteries and the great vessels (Figure 1, Figure 2 and Figure 3).

## 7. Hemodynamic Performance

Hemodynamic performance is a crucial aspect of evaluating the success of the Ross procedure. Studies have shown favorable hemodynamic outcomes in patients who have undergone the procedure. The pulmonary autograft, when used as an aortic valve replacement, has demonstrated excellent hemodynamic properties, including low transvalvular gradients, larger effective orifice areas, and improved left ventricular function [1,3]. These findings indicate that the Ross procedure provides favorable hemodynamic outcomes and contributes to optimal cardiac function.

## 8. Quality of Life and Functional Outcomes

### 8.1. Postoperative Quality of Life Measures

Several studies have investigated the impact of the Ross procedure on postoperative quality of life. These studies have utilized various validated instruments, such as the SF-36 Health Survey and the EuroQol-5D, to assess physical, emotional, and social well-being. Overall, these studies have consistently reported favorable postoperative quality of life outcomes in patients who have undergone the Ross procedure. Patients often experience improvements in symptoms, functional capacity, and overall satisfaction with their cardiac health [43,44].

### 8.2. Assessment of Functional Outcomes and Exercise Capacity

Functional outcomes and exercise capacity are important indicators of the success of the Ross procedure. Studies evaluating these aspects have shown that patients who undergo the Ross procedure often exhibit excellent functional outcomes and have the ability to engage in regular physical activities. Exercise stress testing, including peak oxygen consumption (VO_2_ max) measurements, has demonstrated good exercise capacity in these patients, comparable to or even superior to other valve replacement options [44]. While most valve prostheses will have an increase in AV gradient during exercise, the autograft exhibits hemodynamic characteristics similar to normal human AV even under conditions of enhanced cardiac output [45].

### 8.3. Comparison of Quality of Life and Functional Outcomes with Alternative Procedures

When comparing the quality of life and functional outcomes of the Ross procedure with alternative procedures, such as mechanical valve replacement and bioprosthetic valve replacement, several factors need to be considered. Mechanical valve replacement, while durable, may impact the quality of life and restrict certain activities. Bioprosthetic valve replacement avoids the need for anticoagulation but has limitations in terms of durability and hence the need for future re-intervention. Comparative studies have shown that the Ross procedure can provide similar or superior quality of life and functional outcomes compared to alternative procedures.

## 9. Patient Selection and Risk Stratification

Optimal patient selection is crucial for achieving successful outcomes with the Ross procedure. Several patient characteristics have been identified as favorable for considering the Ross procedure. These include a younger age, an absence of significant coronary artery disease, an absence of aortic root dilatation with aortic stenosis, and an absence of significant left ventricular dysfunction. Patients with these characteristics are more likely to benefit from the Ross procedure due to their potential for long-term durability and improved quality of life [18,46]. Table 2 summarizes candidates that do better and do worse.

Studies evaluating the outcomes and long-term follow-up of the Ross procedure in pediatric and young adult patients have reported promising results. These studies have demonstrated excellent survival rates, favorable hemodynamic performance, and low rates of reoperation. Additionally, long-term follow-up studies have shown low incidences of valve-related complications such as neoaortic regurgitation and neoaortic root dilatation [47,48].

Performing the Ross procedure in pediatric patients including infants and neonates presents unique challenges and considerations. The smaller size of pediatric hearts requires the adaptation of surgical techniques and the use of appropriately sized homografts to replace the used pulmonary valve. Careful assessment of the aortic annulus and root dimensions is crucial to ensure proper matching of the autograft and the aortic root. Usually, under such circumstances, the autograft is larger than the aortic root as the most common lesion in this age group is aortic stenosis with a generally smaller aortic annulus. Enlarging the aortic annulus should be done just enough to match the size of the autograft. The autograft will grow with the child’s growth and most of the time match the somatic growth [49]. Ongoing growth and development necessitate long-term follow-up as reoperation to change the pulmonary valve substitute is inevitable.

The management of pediatric patients also requires multidisciplinary collaboration involving pediatric cardiac surgeons, pediatric cardiologists, and other specialists experienced in treating congenital heart diseases. Preoperative evaluation and risk stratification, as well as postoperative care and follow-up, should be tailored to the unique needs of pediatric patients.

While the Ross procedure has demonstrated favorable outcomes in the pediatric and young adult population, further studies with larger cohorts and longer follow-up are needed to assess the durability and long-term benefits in this specific patient group.

Aortic valve pathology and hemodynamic manifestations are important in selecting patients for the Ross procedure. Patients with dilated aortic roots and pure aortic valve regurgitation do not do as well, particularly if this is related to rheumatic heart disease and associated with mitral valve involvement. Patients with aortic valve disease related to connective tissue disorders are generally not good candidates for the procedure [50].

### The Ross Procedure for the Treatment of Infective Endocarditis

Traditionally, management of infective endocarditis (IE) has involved the utilization of biologic homografts or mechanical valves. However, the Ross procedure emerges as a viable alternative for young adults. It offers a reduced reoperation incidence compared to biologic homografts and eliminates the need for anticoagulation required by mechanical valves—a significant consideration for younger patients, who may exhibit lower compliance. In the absence of randomized controlled trials, small clinical series provide the most reliable evidence. An early series of 28 patients [51], where 14 of the cases were emergency surgeries, reported an in-hospital mortality of 10.7%. The 10-year survival stood at 47%, with three cases of recurrent IE documented. Another cohort of 20 patients [52], including 10 with bicuspid aortic valves, showed only one early mortality and no IE recurrence over a 47-month follow-up, with stable postoperative hemodynamics. A more recent series by Loobuyck et al. [53] encompassed 38 patients with a mean age of 33.9 years, yielding an in-hospital mortality of 5.3% and an overall survival of 82%. Recurrent IE was observed in two patients, and six required reinterventions due to autograft or homograft failure. Despite the limited evidence from small clinical series, the procedure’s favorable outcomes are underscored by relatively low in-hospital mortality and a decent long-term survival rate. Nevertheless, there remains the need for larger, randomized studies to further validate these findings and establish more definitive treatment guidelines for this population.

## 10. Contemporary Techniques and Innovations

In recent years, there have been significant advances in cardiac surgical techniques, including the development of minimally invasive, endoscopic and robotic approaches. Such techniques aim to reduce surgical trauma, shorten the hospital stay and enhance post-operative recovery. These approaches may be utilized in performing the Ross procedure, though this is a complex surgical procedure requiring special expertise. Nevertheless, potential benefits for patients undergoing the Ross procedure are there [54].

Tissue engineering is another area of innovation in cardiac surgery. Researchers are exploring strategies to develop bioengineered grafts and valves using a combination of synthetic materials and patient-specific cells. These bioengineered constructs have the potential to improve the durability and functionality of the pulmonary valve substitutes after the Ross procedure, reducing the need for reoperations and long-term complications [55,56]. Additionally, development of durable patch material may result in improvements in aortic valve repair and reconstruction techniques including the neo-cuspidization (Ozaki) procedure reducing the need for aortic valve replacement in general [57].

Future directions in valve surgery include the exploration of tissue-engineered scaffolds and valve substitutes and the integration of regenerative medicine approaches, aiming to further enhance the outcomes and long-term success and ultimately improving the quality of life of patients requiring a valve replacement.

Ongoing research in the field of the Ross procedure focuses on several areas. Researchers are investigating novel imaging techniques, such as 3D echocardiography and cardiac magnetic resonance imaging, to improve preoperative planning, intraoperative guidance, and postoperative follow-up [58]. Additionally, long-term studies are being conducted to evaluate the durability and outcomes of contemporary Ross procedures, particularly in high-risk patient populations [25,59].

## 11. Conclusions

In conclusion, the Ross procedure, one of the most scrutinized surgical procedures in literature, has emerged as a viable excellent option for aortic valve replacement offering unique advantages in certain patient populations. The pulmonary autograft as an aortic valve substitute is probably currently the closest to an ideal valve substitute. It provides excellent long-term survival matching the normal population, favorable and durable hemodynamic performance and proven potential for growth in the pediatric age group, including neonates and infants (Table 3).

The strength of evidence supporting the benefits of the Ross procedure comes from a combination of retrospective observational studies, prospective registries, and meta-analysis. While the level of evidence varies between studies, there is a consistent trend showing favorable outcomes. Nevertheless, direct head-to-head randomized controlled trials comparing the Ross procedure to alternative aortic valve replacement options are limited. It is important, however, to acknowledge that the procedure does have some limitations. Further research, including well-designed prospective studies, is needed to validate and strengthen the current evidence base.

The Ross procedure should be considered a valuable option in the armamentarium of adult and congenital cardiac surgeons. Careful selection is of utmost importance for a successful long-lasting outcome and should be individualized based on the patient’s characteristics and preferences.

In that regard, we support “a hard look at current practices and a call for re-evaluation of the current guidelines” [60,61].

## Figures and Tables

**Table 1:** Short- and long-term outcomes of the Ross procedure in different studies. N denote number of patients, date denote date of study publication.

Relevant Study	Ross Procedure Outcomes
El-Hamamsy et al., RCT n(228) 2010 [9]	10 Year Survival 97%
Ryan et al., 2021 n(225) [26]	20 Year Survival 81.3% (74.8–88.3%)
	Mortality (In-hospital) 0.9% (30 day) 2.2%
Pergola et al., n(536) 2020 [18]	15 Year Freedom from all Re-operation 83%
	Freedom from Autograft reoperation 81%
David et al., n(212) 2019 [23]	20 Year Mortality 10.8%
Stelzer et al., n(702) 2021 [25]	Perioperative Mortality 1%
Aboud et al., n(2444) 2021 [28]	25 Year Survival 75.8%
	Early mortality 1%

**Table 2:** Summarizing patient who will do better or worse after the Ross procedure.

Candidate Selection	
Better	Worse
Congenital etiology	Rheumatic
Aortic stenosis	Pure Aortic regurgitation
Aortic root diameter < 15 mm/m^2^	Older age (homograft re-intervention)

**Table 3:** Table demonstrating the characteristics of the autograft as an ideal valve. the number of +++ denotes more benefits relative other procedures.

Features	Benefit
Silent	++++
Non-thrombogenic	++++
Normal Hemodynamic	++++
Readily Available–Low cost	++++
Has Potential for growth	+++
Infection Resistant	+++
Easy to Implant	++
Durable-Non-Rheumatics -Rheumatics	++++ ++

## Data Availability

Not applicable.
